# Use of Educational Technology in Inclusive Primary Education: Protocol for a Systematic Review

**DOI:** 10.2196/65045

**Published:** 2025-02-25

**Authors:** Erica Ranzato, Catherine Holloway, Maryam Bandukda

**Affiliations:** 1 Department of Computer Science University College London London United Kingdom

**Keywords:** special education needs, disabilities, primary education, inclusive education, education technology, assistive technology, high-income countries, systematic review

## Abstract

**Background:**

Educational technology (EdTech) has been instrumental in the last few decades in promoting inclusive education by overcoming various learning barriers and offering tools and opportunities to all students, including those with special educational needs and disabilities (SEND). However, there is limited understanding of current classroom practices and policies and of the effects of the COVID-19 pandemic on EdTech use in the inclusive classroom.

**Objective:**

This systematic review aims to outline the current knowledge on the use of EdTech to support the learning of students with SEND in inclusive primary schools in high-income countries.

**Methods:**

We followed the PRISMA-P (Preferred Reporting Items for Systematic Review and Meta-Analysis Protocols) and the Generalized Systematic Review Registration Form in reporting the details of this protocol. The inclusion criteria for the systematic review require that studies focus on students with SEND who are attending the primary stage of school in high-income countries. The studies can be qualitative or quantitative and should explore the design and use of EdTech with these students. Eligible studies must be published between 2016 and 2024, be peer-reviewed, and be available in English. We systematically searched the ACM, Directory of Open Access Journals, British Educational Index, ERIC, Google Scholar (first 100 records), IEEE, PsycINFO, Scopus, and Web of Science databases. The titles and abstracts of all records will be screened for relevance according to the inclusion criteria. Following this, the full text of the articles will be screened. To ensure the reliability of the screening process, an independent reviewer will screen a percentage of the records for the first screening round. The data extraction process for this systematic review will start with a pilot stage to validate and eventually update the list of entities to be extracted. Following the pilot stage, the final data extraction will be undertaken. An independent reviewer will extract data from a subsample of the records to ensure the reliability of the data extraction process.

**Results:**

The database search was conducted in July 2024. The database search identified a total of 547 records. It is anticipated that the study findings will be submitted for publication in a peer-reviewed journal by the end of January 2025.

**Conclusions:**

This study will provide up-to-date evidence of the use of EdTech in inclusive primary school settings in high-income countries and will describe the impact of the COVID-19 pandemic on the use of EdTech with students with SEND.

**International Registered Report Identifier (IRRID):**

DERR1-10.2196/65045

## Introduction

### Background

Educational Technology (EdTech) usually includes a number of broad definitions across disciplines. For the purposes of this systematic review, this term includes any use of information and communication technology and assistive technology. Information and communication technology refers to the technologies used for accessing, processing, and communicating information and encompasses a wide range of technologies, including (1) computers and software applications, (2) internet and network systems, (3) mobile phones and other handheld devices, (4) digital broadcasting technologies (radio and TV), and (5) email and other communication tools [1]. Assistive technology includes any item or piece of equipment that helps a person with a disability to increase, maintain, or improve their functional capabilities as a learner and any related assistive technology service [1].

EdTech has transformed education and learning in the last few decades by offering multiple means to represent information, express knowledge, and engage in learning [2]. Moreover, EdTech has played a crucial role in supporting inclusive education by addressing multiple barriers to learning and by providing tools and opportunities to all students, including students with special educational needs and disabilities (SEND). For instance, EdTech has supported fair and optimized access to the curriculum while developing students’ independence, agency, and social inclusion [2]. In addition, it has facilitated personalized learning, enhanced communication, and interaction among peers and teachers, and strengthened social skills [2]. However, as noted in the UNESCO (United Nations Educational, Scientific and Cultural Organization) report [2], the overall benefits and drawbacks of the implementation of EdTech are still not fully understood. Several factors contribute to this lack of understanding. First, the effectiveness of EdTech tools varies by the socioeconomic level of the students and by the income level of the country. It also depends on teacher willingness to adopt these tools and their readiness to use them as well as on the education stage. In fact, students at different stages show distinct behavior habits in terms of web-based learning experiences [3]. Third, the costs associated with implementing and maintaining EdTech, both in the short term and in the long term, may be higher than initially anticipated, posing affordability challenges, especially in poorer countries. These costs include the cost of the EdTech tools as well as the cost of training teaching staff members, which is necessary for the effective use and the appropriate selection of technologies for specific students. Finally, not all technologies are suitable for students with different types of SEND. To be effective, these technologies must be tailored to each student’s specific learning needs.

With such numerous factors affecting the effectiveness of EdTech and the wide range of software applications, devices, and other technologies available on the market, teachers and policy makers can easily feel overwhelmed. Therefore, systematic reviews are needed to understand the available options and to help them make informed decisions for effective and manageable employment of EdTech in education to facilitate the inclusion of students with SEND. In recent years, a few systematic reviews have examined the use of EdTech for students with SEND [1,4-6]. Despite the importance of these studies, some are limited in their scope and in the variety of EdTech tools examined, while others encompass all educational levels, preventing a focused analysis of EdTech effectiveness for specific educational stages. In addition, only one study included research published after the COVID-19 pandemic.

The systematic review by [4] investigated the technology practices in special education contexts and included 126 studies published between 2014 and 2018. The results revealed that the most examined technology was games, and the most studied outcome was the improvement of learners’ cognitive abilities. The majority of the studies included precollege students with learning disabilities and focused on natural sciences. Interventions were primarily conducted in formal educational environments and were mainly implemented over 5-10 weeks. Based on their results, the authors recommended providing a greater level of detail in reporting research findings and placing more emphasis on promoting life, job, and social skills.

The study by [5] investigated the impact of using augmented reality in the education of students with SEND. The review included 18 studies published between 2016 and 2021. The authors noted a decrease in studies from 2020 onwards, which they attributed to the COVID-19 pandemic and to the consequent closure of educational centers. The majority of the included studies used quantitative methodologies. Most of these studies focused on primary and secondary school students, with very few examining early childhood education or higher education. The experiences were primarily conducted with students with intellectual disabilities, followed by students with autism, learning difficulties, and hearing impairments. The use of augmented reality showed positive results in the learning of students with SEND. Improvements were observed in academic performance, motivation, communication, social interaction, and level of autonomy. However, the authors identified several limitations to the use of augmented reality in the education of SEND. These included low levels of teacher training, limited availability of augmented reality technology, lack of support from educational institutions, and technical and accessibility issues.

The study by [6] presented a systematic review investigating technology-enhanced and game-based learning activities used with children with SEND. The authors included 18 studies published between 2009 and 2019. They reported that about one-third of the studies involved participants with intellectual disabilities, another third included autistic participants, and the remaining studies involved participants with Down syndrome, motor impairments, visual impairments, and hearing impairments. The primary goal of the game-based activities was to support students’ cognitive skills. These activities covered a wide range of academic areas, including mathematics, functional skills, and communication. The results of the included studies were mixed, with some interventions improving the learning of participants and others promoting their motivation.

Finally, the systematic review by [1] investigated the use of assistive technology among primary school students with disabilities in low- and middle-income countries. The study included 51 studies published between 2007 and 2020. The findings showed little evidence of the efficacy of educational interventions, with learning outcomes often considered secondary to the technological aspects of the studies. The authors reported a considerable variation in the number of studies addressing different types of impairments, with two-thirds of the studies involving students with sensory impairments. In addition, teachers and parents were often excluded from the process of using and evaluating EdTech. A consistent theme reported in the included studies was the reluctance of teachers to adopt EdTech solutions in their everyday teaching practices. Most of the studies were case studies or small-sample multiple baseline studies and rarely included control groups.

The current systematic review aims to summarize the current understanding of how EdTech supports the learning of students with SEND in inclusive primary schools in high-income countries. Moreover, this study will allow the investigation of the effects of the COVID-19 pandemic on the use of EdTech in this specific setting.

### Review Questions

This systematic review seeks to answer the following questions:

1. How can EdTech support students with SEND in primary school settings in high-income countries?
2. What EdTech interventions are used to support students with SEND in high-income countries?
3. What are the gaps in the literature, and what is the potential for further development in this field?
4. How has the COVID-19 pandemic impacted the use of EdTech in primary inclusive classrooms?

## Methods

### Study Registration

The protocol for this systematic review follows the PRISMA-P (Preferred Reporting Items for Systematic Review and Meta-Analysis Protocols) checklist [7,8] and the Generalized Systematic Review Registration Form [9]. The PRISMA-P checklist used for the development of this systematic review protocol is provided in Multimedia Appendix 1. The systematic review protocol was preregistered before the analysis of the data in the Open Science Framework in July 2024 and was last updated in December 2024 [10].

### Inclusion Criteria

To be included in the systematic review, studies must meet the following criteria.

#### Population

- Students with SEND.
- Attending primary school.

#### Setting

We will include studies conducted in high-income countries, as classified by the World Bank [11] and based on the World Bank’s Gross National Income per capita thresholds for the 2024-2025 fiscal year.

#### Study Design

We will include qualitative and quantitative studies exploring the design and the use of EdTech tools with students with SEND. We will exclude reviews of the literature.

#### Publication Date

We will include studies published between 2016 and 2024. This timeframe encompasses 4 years before the COVID-19 pandemic, which provides a baseline, and 4 years after, allowing us to assess the immediate and evolving effects of the pandemic. Moreover, focusing on this timeframe complements existing literature, which already covers earlier years, and provides a comprehensive and up-to-date perspective on the subject.

#### Publication Type

We will include peer-reviewed journal articles and peer-reviewed conference proceedings. While we originally considered including gray literature, such as reports from nongovernmental organizations, we realized that these sources would require a different approach to data extraction and synthesis, demanding additional time and resources that are not available to our research team. Similarly, Master and PhD theses will be excluded. Although these documents can be valuable resources, they may not be subjected to the same level of scrutiny as peer-reviewed articles, depending on the country in which they are conducted. Their length and the challenge of assessing them raise concerns about data extraction and synthesis of the findings for the systematic review. Moreover, their limited accessibility and dissemination could complicate replicability and comprehensive data extraction. Finally, we assumed that high quality studies are typically published in a peer-reviewed journal, supporting our decision to exclude these sources.

#### Language

We will include only studies available in the English language.

### Exclusion Criteria

Studies that do not involve participants with SEND will be excluded.

In addition, studies focusing on individuals outside the primary school age range (5 years to 11 years) will not be considered. This exclusion criterion will be based on the mean age of the sample of students with SEND, which has to fall within this range.

Research conducted in countries other than high-income nations, as classified by the World Bank [11], will also be excluded.

Studies conducted in contexts other than the classroom settings, such as hospitals, clinical settings, or participants’ homes will be excluded.

Furthermore, non–peer-reviewed studies and gray literature will be excluded. Previous reviews of literature will also be excluded.

Finally, studies not available in English will be excluded.

### Search Strategy

Literature search strategies were developed using keywords from previous relevant systematic reviews, with guidance from a specialist librarian at UCL. The librarian provided recommendations on both the keyword list and database selection. The databases searched include ACM, Directory of Open Access Journals, British Educational Index, ERIC, Google Scholar (first 100 records), IEEE, PsycINFO, Scopus, and Web of Science. To ensure comprehensive coverage, the electronic database search will be supplemented by scanning the reference lists of studies that meet the inclusion criteria.

Starting from the list of keywords used by [1], the list of keywords was optimized by including synonyms and relevant terms derived from the research questions. The search strategy underwent several iterations to refine the terms and optimize the query string. The desirability of reducing the number of keywords or adding new terms to the query was evaluated based on the effect on the number of hits [12]. The terms evaluated were not considered necessary and hence deleted if the number of hits increased greatly and included a high ratio of nonrelevant references. To further validate the search results, we ran the search using alternative search terms to confirm that the search string captured the relevant studies. Search terms were limited to the title, the abstract, and the keywords of the papers, when possible. Alternatively, they were limited to the abstract of the paper only. When possible, the search was restricted to specific subject areas (such as “psychology” or “education”) or educational levels (such as “primary school”). The search strategy was conducted on July 26, 2024, by ER. Table 1 displays the search strategy for each database.

**Table 1:** Search strategy formulated for each database included in the study.

Database	Search string
PsycINFO^a^	(blind or deaf* or autis* or neurodiver* or “intellectual dis*” or “learning dis*” or “mental* retard*”).mp. [mp=title, abstract, heading word, table of contents, key concepts, original title, tests & measures, mesh word] AND (tech* or assistive or smartphone or tablet or laptop).mp. [mp=title, abstract, heading word, table of contents, key concepts, original title, tests & measures, mesh word] AND (“primary school” or “elementary school” or “junior school” or “middle school”).mp. [mp=title, abstract, heading word, table of contents, key concepts, original title, tests & measures, mesh word] Filters applied: limit to (english language and abstracts and 180 school age <age 6 to 12 yrs> and journal article and yr=“2016 - 2024”)
Web of Science	(((TS=(blind OR deaf* OR autis* OR neurodiver* OR “intellectual dis*” OR “learning dis*” OR “mental* retard*” )) AND TS=(tech* OR assistive OR smartphone OR tablet OR laptop)) AND TS=(“primary school” OR “elementary school” OR ”junior school“ OR ”middle school“)) AND (DT==(”ARTICLE“) AND TASCA==(”EDUCATION SPECIAL“ OR ”PSYCHOLOGY EDUCATIONAL“ OR ”EDUCATION EDUCATIONAL RESEARCH“) AND LA==(”ENGLISH“) AND PY=2016-2024)
Scopus	( TITLE-ABS-KEY ( blind OR deaf* OR autis* OR neurodiver* OR ”intellectual dis*“ OR ”learning dis*“ OR ”mental* retard*“) AND PUBYEAR > 2015 ) AND ( TITLE-ABS-KEY ( tech* OR assistive OR smartphone OR tablet OR laptop ) AND PUBYEAR > 2015 ) AND ( TITLE-ABS-KEY ( ”primary school“ OR ”elementary school“ OR ”junior school“ OR ”middle school“ ) AND PUBYEAR > 2015 ) AND LANGUAGE ( english ) AND SUBJAREA ( psyc OR soci-edu OR comp-csa OR comp-hci ) AND ( LIMIT-TO ( DOCTYPE,”ar“ ) OR LIMIT-TO ( DOCTYPE,”cp“))
ERIC^b^	Noft(blind OR deaf* OR autis* OR neurodiver* OR ”intellectual dis*“ OR ”learning dis*“ OR ”mental* retard*“) AND noft(tech* OR assistive OR smartphone OR tablet OR laptop) AND noft(”primary school“ OR ”elementary school“ OR ”junior school“ OR ”middle school“) AND rtype.Exact(”Article“) AND la.Exact(”English“) AND lv(”primary education“ OR ”elementary education“ OR ”middle schools“) AND PEER(yes) AND PEER(yes) AND pd(20160101-20240630) Filters applied: Document type: Article; Language: English; Education level: Elementary education, Middle schools, Primary education
British Education Index	AB ( blind OR deaf* OR autis* OR neurodiver* OR “intellectual dis*” OR “learning dis*” OR “mental* retard*” ) AND AB ( tech* OR assistive OR smartphone OR tablet OR laptop ) AND AB ( ”primary school“ OR ”elementary school“ OR ”junior school“ OR ”middle school“ ) Limiters: Peer Reviewed; Publication Date: 20160101-20241231; Publication Type: Academic Journal; Language: English Expanders: Apply equivalent subjects Search modes: Proximity
Directory of Open Access Journals	blind OR deaf* OR autis* OR neurodiver* OR intellectual dis* OR learning dis* OR mental* retard* AND tech* OR assistive OR smartphone OR tablet OR laptop Filters applied: 2016-2024
ACM^c^ Digital Library	[[Abstract: blind] OR [Abstract: deaf*] OR [Abstract: autis*] OR [Abstract: neurodiver*] OR [Abstract: ”intellectual dis*“] OR [Abstract: ”learning dis*“] OR [Abstract: ”mental* retard*“]] AND [[Abstract: tech*] OR [Abstract: assistive] OR [Abstract: smartphone] OR [Abstract: tablet] OR [Abstract: laptop]] AND [[Abstract: ”primary school“] OR [Abstract: ”elementary school“] OR [Abstract: ”junior school“] OR [Abstract: ”middle school“]] AND [E Publication Date: (01/01/2016 TO 30/06/2024)]
IEEE^d^ Xplore	(“Abstract”: blind OR “Abstract”: deaf* OR “Abstract”: autis* OR “Abstract”: neurodiver* OR “Abstract”: “intellectual dis*” OR “Abstract”: “learning dis*” OR “Abstract”: “mental retard*”) AND (“Abstract”: tech* OR “Abstract”: assistive OR “Abstract”: smartphone OR “Abstract”: tablet OR “Abstract”: laptop) AND (OR “Abstract”: “primary school” OR “Abstract”: “elementary school” OR “Abstract”: “junior school” OR “Abstract”: “middle school”). Filters applied: 2016-2024 & Journals & Conferences
Google Scholar	(blind OR deaf* OR autis* OR neurodiver* OR “intellectual dis*” OR “learning dis*” OR “mental* retard*”) AND (tech* OR assistive OR smartphone OR tablet OR laptop) AND (”primary school“ OR ”elementary school“ OR ”junior school“ OR ”middle school“) Filters applied: 2016-2024

^a^PsycINFO: American Psychological Association.

^b^ERIC: educational resources information center.

^c^ACM: association computing machinery.

^d^IEEE: Institute of Electrical and Electronics Engineers.

### Screening

Rayyan [13], a web-based literature review software, will be used to remove the duplicates and to assist with the screening process. The titles and abstracts of all the records will be screened for relevance against the inclusion criteria by ER. Following this, the same reviewer will evaluate the full text of the articles deemed eligible. The reference list of all the studies that ultimately will meet inclusion criteria from the database search will be screened for any additional records that may have been missed.

An independent reviewer will screen a subsample of all records to establish the reliability of the screening process. The percentage of records to be independently screened will be determined based on the total number of records, according to the following rule: if there are more than 201 records, 5% will be screened; if there are between 100 and 200 records, 10% will be screened; and if there are 100 or fewer records, 20% will be screened. Disagreements will be discussed and resolved during agreement meetings through consensus or by consulting a third reviewer. The rationale for excluding records that do not meet the eligibility criteria will be documented.

### Data Extraction

The data extraction process will begin with a pilot stage, during which ER and MB will independently extract data from a randomly selected sample of the included studies. The sample size will be determined based on the total number of studies included at this stage of the review, following the same criteria used for the screening process. The purpose of the pilot phase is to ensure adherence to standardized procedures and to validate and, if necessary, update the data extraction criteria. After the pilot stage, ER will complete the final data extraction for all included studies, using Microsoft Excel to collect and organize the information.

The provisional list provided in Textbox 1 outlines the data to be extracted. Based on the pilot stage, this list and the corresponding coding rules will be finalized. If significant updates are made to the list of entities to be extracted, MB will independently review the data extraction to ensure the reliability of the final data extraction process. The sample size will be determined based on the total number of studies included at this stage of the review, following the same criteria used for the screening process.

The study’s characteristics include the authors, publication year, and the country of data collection. It also includes the study design (cross-sectional or longitudinal) and type of study based on the category used in the Mixed Methods Appraisal Tool (MMAT) [14]. For interventions and single-case research, information about the length and framing of the intervention will also be extracted. The intervention’s framing will be coded based on the Multi-Tiered System of Support model, which operationalizes how teachers implement the Universal Design for Learning model and includes three categories: (1) core classroom instruction, (2) targeted small group instruction, or (3) intensive individual intervention [15].

The SEND sample characteristics cover sample size, participant age, sex, socioeconomic status, and the diagnosis or disability categorization.

EdTech characteristics include the terminology used to refer to the tool described in the study (eg, “information and communication technology” or “assistive technology”), cost, level of technology (low-tech or high-tech), and the type of EdTech tools used, categorized according to the World Bank [16], as shown in Textbox 2.

**Provisional list of data to be extracted**

**Characteristics of the study**

- Authors
- Year of publication
- Country where the data was collected
- Study design
- Type of study
- Length and framing of the intervention (limited to intervention studies and single-case research)

**Characteristics of the sample with SEND**

- Sample size
- Chronological age of participants
- Sex of participants
- Socioeconomic status
- Diagnosis or disability categorisation

**Characteristics of Educational Technology (EdTech)**

- Use of EdTech terminology
- Overall cost of the EdTech tool
- Level of technology of the EdTech tool
- Type of EdTech employed

**Type of education technology (EdTech).**

**Type of EdTech**

- Augmentative and alternative communication
- Accessible textbooks
- Assistive hearing and listening technology
- Braille reading and writing equipment
- Mainstream accessible software and applications
- Mobility technology
- Personal electronic devices
- Platforms and applications for learning support
- Technology for teaching support
- Technology for vision enhancement
- Text-to-speech technology

### Outcomes

The primary outcome of this systematic review will be the overall effectiveness of EdTech tools used in the inclusive classroom, defined as their impact on a range of factors including academic achievement, student engagement, social inclusion, accessibility, and other relevant outcomes that contribute to an inclusive learning environment.

#### Assessment of Risk of Bias in the Included Studies

To assess the risk of bias in the studies included in this systematic review, we will use the MMAT [14]. The MMAT is a generic critical appraisal tool that was developed for the appraisal of multiple types of studies including qualitative studies, quantitative randomized controlled trials, quantitative nonrandomized studies, quantitative descriptive studies, and mixed methods studies. As the present systematic review includes studies using a range of methodologies, the MMAT was deemed the most suitable approach for ensuring quality and consistency in the evaluation of the risk of bias. The assessment consists of 2 screening questions and 5 category-specific items. Each item can be rated as “yes” if the criterion is met, “no” if it is not met, or “can’t tell” if the study provides insufficient information to score the criterion as met or not met. The MMAT has been applied in previous research, such as in systematic reviews to assess the methodological quality of the included studies investigating the use of EdTech for health and social care practitioners and the use of gamification in education [17,18].

All included studies will be evaluated for quality by one reviewer of the research team. In addition, an independent reviewer will reassess 20% of those studies to ensure consistency and reliability in the evaluation process. Disagreements will be resolved through discussion, and if consensus cannot be reached, a third reviewer will be consulted.

#### Data Synthesis

We will present the collated results in a structured format, aligned with the research questions of the systematic review. This review will use a mixed methods analysis, incorporating both quantitative and qualitative approaches to analyze the results from the included studies.

For the interventions, we will assess the primary outcome by comparing pre- and posttest scores of students with SEND using specific EdTech tools. Tables will be created to describe the characteristics of the studies, populations, and EdTech tools used.

Qualitative data will be analyzed using content analysis to synthesize and interpret key issues and themes related to Review Question 1 across the studies. If a sufficient number of comparable studies are included, a subgroup analysis will be conducted. Potential subgroups may be based on the type of EdTech used, the population involved, or the country where the study was conducted.

#### Assessment of Meta-Biases

We anticipate that assessing publication bias may not be feasible due to the heterogeneous nature of the included studies. Similarly, assessing outcome reporting bias may be challenging because of the potential lack of registered protocols for these studies. However, we will provide a narrative discussion on the potential for publication bias, focusing on whether negative results were excluded or difficult to identify, and we will highlight any inconsistencies in outcome reporting where possible.

## Results

**Figure 1:**
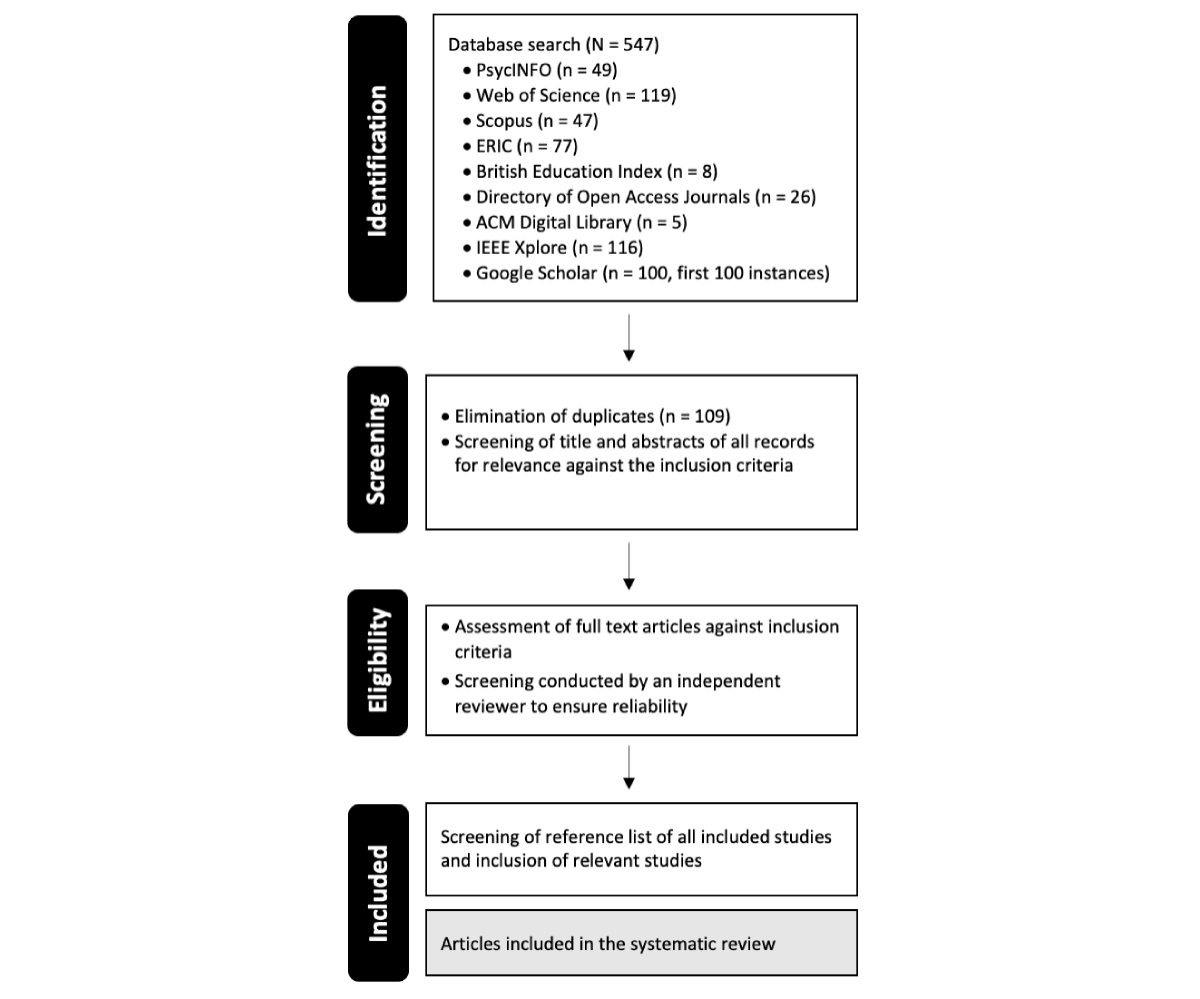
Flow diagram of the search process PsycINFO: American psychological association; ERIC: educational resources information center; ACM: association computing machinery; IEEE: Institute of Electrical and Electronics Engineers.

As shown in Figure 1, a database search was conducted in July 2024, identifying 547 records. We expect to complete the screening process by November 2024 and submit the manuscript for peer review by the end of January 2025. The results of the completed study will be submitted to a journal relevant to the SEND population, and findings will be presented at relevant conferences and shared with stakeholders, such as nongovernmental organizations, education and health care organizations, assistive technology developers, and policy makers.

## Discussion

### Principal Findings

Many reviews have already been conducted on the use of EdTech with students with SEND. However, these reviews are limited by their focus on specific tools, such as augmented reality or educational game-based activities. In addition, some reviews encompass all educational levels, which prevents a detailed examination of the effectiveness of these tools for specific educational stages (such as primary, secondary, or higher education). These limitations reduce their usefulness for making targeted decisions about EdTech implementation. Furthermore, some reviews have a narrow scope regarding publication years, covering studies only up to 2021. It is essential to have current insights into classroom practices, as technology and devices used just 5 years ago may now be outdated or unavailable, and new EdTech devices may have been introduced and evaluated. To fill this gap, our systematic review will collect and analyze data on the use of EdTech in inclusive primary school settings in high-income countries and will also discuss findings in comparison with previous reviews of literature in this field. Finally, this study will describe the impact of the COVID-19 pandemic on the use of EdTech in the field of research education. We anticipate that the review will reveal an increase in the use of EdTech in the inclusive classroom following the COVID-19 pandemic. These findings are expected to inform the use and the design of inclusive EdTech and assistive technology tools to support students with SEND in the primary classroom and to establish best practices for the seamless integration of EdTech, ultimately producing positive outcomes for these students.

In addition, one of the outcomes of our systematic review will be the creation of an updated database that catalogs all studies investigating the use of EdTech in inclusive primary schools in high-income countries. This database will offer accessible, current evidence on how EdTech supports students with SEND in this setting. The final database, which will be published in the Open Science Framework platform and as supplementary material following the publication of the systematic review, aims to facilitate evidence-based decision-making, support the development of guidelines, interventions, and policies, and identify gaps in the current research landscape. It is expected to serve as a valuable resource for researchers, educators, assistive technology developers, policy makers, and other stakeholders.

### Limitations

While we will use a comprehensive search strategy, it is possible that relevant studies could be overlooked. In addition, our search is restricted to English-language and peer-reviewed publications, potentially excluding studies in other languages and unaudited studies and thereby limiting the comprehensiveness of the evidence available. Finally, a limitation of this systematic review is that it may not be feasible to meta-biases. As a result, the confidence in the conclusions drawn from this review may be somewhat limited, and this limitation should be considered when interpreting the findings.

### Conclusions

To our knowledge, this will be the first systematic review to describe the impact of the COVID-19 pandemic on the use of a wide range of EdTech to support the learning and inclusion of primary school students with SEND in high-income countries. This review aims to facilitate evidence-based decision-making and contribute to the future improvement of guidelines for the effective use of EdTech to support inclusion in primary school settings. Such knowledge is crucial for promoting educational equity, enhancing learning outcomes, and supporting the diverse needs of all students.

## Appendix

Multimedia Appendix 1 PRISMA-P (Preferred Reporting Items for Systematic Reviews and Meta-Analysis Protocols) checklist.

## Abbreviations

EdTech: educational technology
MMAT: Mixed Methods appraisal Tool
PRISMA-P: Preferred Reporting Items for Systematic Reviews and Meta-Analyses Protocols
SEND: special educational needs and disabilities
UNESCO: United Nations Educational, Scientific and Cultural Organization

## Data Availability

The dataset generated during this study is available in the OSF repository [10] and will be published as supplementary material alongside the main systematic review paper.
